# Numerical Simulation of the Shear Capacity of a GFRP-Strengthened Natural Bamboo-Bolt Composite Joint

**DOI:** 10.3390/polym14153024

**Published:** 2022-07-26

**Authors:** Quanfeng Li, Xiaodong Ji, Zihao Jin, Jin Xu, Shihan Yang, Shumin Lv

**Affiliations:** School of Soil and Water Conservation, Beijing Forestry University, Beijing 100083, China; liquanfengbjfu@163.com (Q.L.); jinzihao@bjfu.edu.cn (Z.J.); xj13146071866@bjfu.edu.cn (J.X.); y199992@bjfu.edu.cn (S.Y.); lvshumin@bjfu.edu.cn (S.L.)

**Keywords:** bamboo, composite joint, numerical simulation, European yield model

## Abstract

As an ecological green building material, natural bamboo has many advantages such as a light weight, high strength, and short growth cycle. Natural bamboo is widely used in landscape architecture and fabricated structures. However, in bamboo building structures, the most common bolted joints often appear cleaved along the grain. In this paper, glass fiber-reinforced polymer (GFRP) is designed to wrap and improve the shear capacity of natural bamboo-bolt composite joints. According to the corresponding material parameters, the finite element model of composite joints is established, and the key influencing variables of the bearing capacity, namely the bolt diameter, bamboo tube outer diameter, and screw end distance, are analyzed. In addition, according to the European analytical yield model of bolted connections, the analytical calculation method of the bearing capacity is proposed and compared with the experimental and simulated values. The results showed that the numerical model and the modified analytical model can suitably describe the bearing capacity of composite joints, and a higher bolt diameter, along with the bamboo outer diameter, will lead to a higher ultimate load of the composite joints. Moreover, the bearing capacity of composite joints has no obvious relationship with the end spacing.

## 1. Introduction

With more attention being drawn to sustainable development and environmental protection, the exploitation of green new materials has become an important trend in civil engineering development [1]. As an ecological composite material, bamboo has advanced renewability and carbon sequestration. Moso bamboo has the strongest carbon sequestration capacity among the subtropical forest species [2], and the life cycle of bamboo products is longer than decades, which can preserve the carbon stored for a long time and delay carbon emissions [3]. Located in the most central distribution place of bamboo in the world, China ranks first in the area, species, stock, and harvesting of bamboo [4]. Bamboo has great development value as a building material, with a short growth cycle of 3–5 years. Moreover, compared with wood and ordinary steel, bamboo has excellent mechanical properties, such as lower density, higher specific strength, and higher specific stiffness; thus, bamboo is also widely used in civil engineering structures [5]. In modern bamboo building structures, the design of the joints is particularly important, and their mechanical properties determine the safety performance of the whole structure [6,7]. Traditional bamboo structure joints mostly adopt rivets and strapping methods, which have complex processing schemes, low construction efficiencies, and insufficient bearing capacities. Therefore, bolted joints are gradually becoming more utilized because of their convenience in the manufacturing process and high bearing capacity. Although bamboo has many excellent mechanical properties, its internal fibers are all along the grain, which leads to weak shear strength and tensile strength in the direction perpendicular to the grain. In particular, natural bamboo culm is easy to split along the grain, thus limiting the broader application of bolt composite joints in bamboo structures [8].

To enhance the bearing capacity of bamboo-bolt composite joints and avoid brittle splitting failure, scholars have tried many different methods. However, most of these methods involve filling up the bamboo tube with other materials, which greatly increases the structural weight and processing complexity. Morisco et al. [9] designed bamboo-bolt composite joints filled with mortar and connected with external steel plates, which greatly increased the bearing capacity but simultaneously increased the weight of the joints by 2.5 times. Hu et al. [10] designed bamboo-bolt composite joints filled with mortar, which increased the tensile capacity by 82%. To elevate the bearing capacity and ductility of the structure without affecting the weight of the structure, many scholars used fiber-reinforced polymers (FRPs) to strengthen the bamboo and wood structures. FRP has excellent characteristics, including a light weight, high strength, and corrosion resistance. It was initially used in aerospace and electronics fields and has been gradually applied in structural reinforcement and repair since the 1990s [11,12]. Among the many kinds of FRP, glass fiber-reinforced polymers (GFRPs) and carbon fiber-reinforced polymers (CFRPs) are mainly used in engineering [13]. Perfetto et al. [14] investigated the structural behavior of short GFRP plates (PA66 GF30) under Low Velocity Impact (LVI) phenomena, which is important in the simulation of the experimental tests on large structural component. Currently, studies on FRP-reinforced bamboo and wood structures mostly concentrate on beams and columns, and little attention is given to joint reinforcement. Davalos et al. [15] reinforced wooden sleepers by wrapping them with GFRP, which improved the bearing capacity and ductility of the structure. Nor et al. [16] wrapped bamboo beams with GFRP, which increased their flexural strength by 100%. Zhang et al. [17] strengthened bamboo tubes by wrapping GFRP so that the axial load capacity increased by 5.5–11.7%. Awaludin et al. [18] designed bamboo-bolt composite joints wrapped with GFRP on the ends, which showed a slight increase in the bearing capacity along with the debonding failure of the GFRP cloth. At the same time, with the development of finite element simulation technology, the application of numerical calculation methods plays an important role in promoting the whole application research of bamboo structures [19]. Wei et al. [20] applied ABAQUS to analyze the flexural performance of bamboo beams reconstituted and reinforced with GFRP, and the simulation results were consistent with the test results, which indicates that the model was effective. Li [21] employed ANSYS to analyze the fatigue performance of composite bamboo columns reinforced with GFRP and showed that GFRPs can effectively improve the fatigue life of the bamboo columns. Donato et al. [22] designed a bamboo treadmill bike through ABAQUS and analyzed its characteristics of stiffness and structural strength, and the results revealed that the main frame made of bamboo could meet specification requirements and actual needs.

Therefore, to enhance the shear capacity of bamboo-bolt composite joints along the grain and prevent brittle splitting failure, a new bamboo-bolt composite joint wrapped and strengthened with GFRP is designed in this paper. The numerical model of the strengthened joints is established by ABAQUS, and the influences on the shear capacity, including the outer diameter and thickness of the bamboo culm wall along with the diameter and position of the bolts, are analyzed. Moreover, the failure mode of the composite joints is investigated based on the calculated results of the numerical model. In addition, a laboratory test is conducted to verify the finite element model, and an analytical method is developed for comparison with the simulation method.

## 2. Geometric Data of the Composite Joint Model and Shear Test Method

The composite joint consists of the following three types of materials: grade 8.8 stud bolts, GFRP sheets, and natural bamboo culms without slubs. The outer wall of the bamboo culm is wrapped with a single layer of the GFRP sheet and tightly bonded together with an epoxy resin structural adhesive. A pair of bolt holes is passed through the outer diameter of the bamboo culm, and the stud bolts are passed through the bolt holes with the ends equal to the outer wall of the bamboo culm.

**Figure 1:**
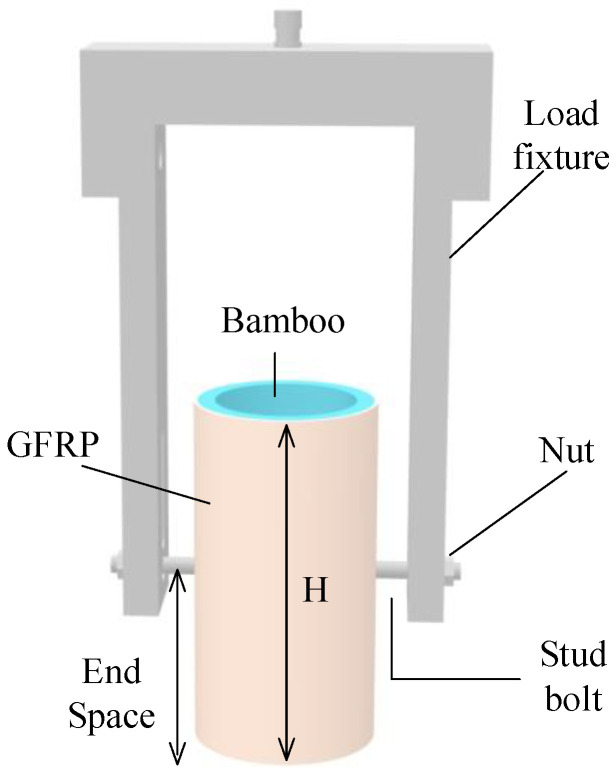
Sketch diagram of D100-1/2H-M10-1.

**Figure 2:**
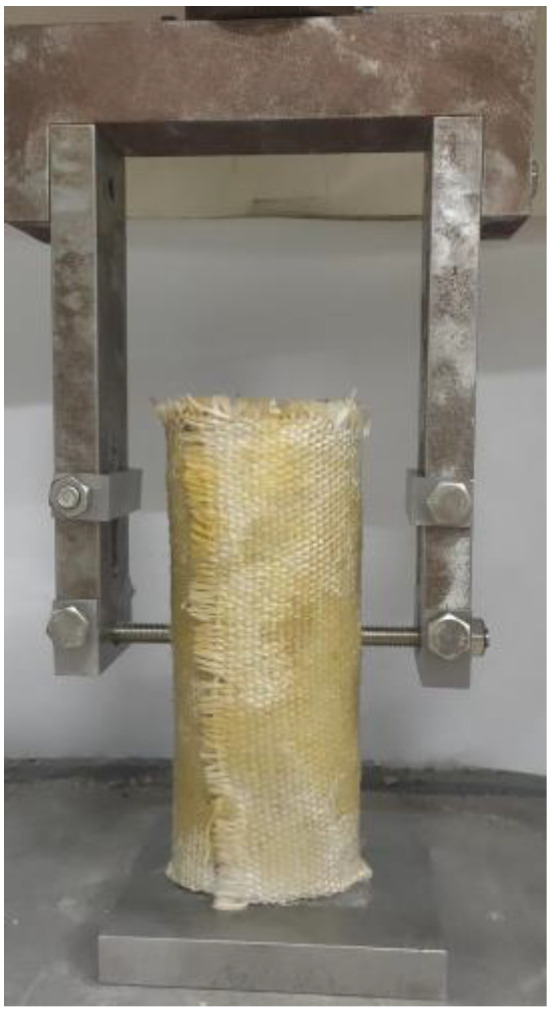
Physical diagram of D100-1/2H-M10-1.

In the test variables considered in this paper, the diameters of the screw holes are 8 mm, 10 mm, and 12 mm. The height of the bamboo culm that is recorded as H is 200 mm, and the edge distance is defined as the distance between the bolt and pressurized end of the bamboo culm, which are H/4, H/2, and 3H/4, respectively. The outer diameters of the bamboo culms are 90 mm, 100 mm, and 110 mm. The wall thicknesses of the bamboo culms are all 10 mm. Considering the change in the outer diameter of the bamboo culm and the length of the fasteners, the length between the fixed ends of the bolts is 140 mm. The nominal rule for joint specimens is as follows: “D outer diameter-end spacing-M screw diameter-number”. The end spacing is defined as the distance between the bolt and the compression end, as shown in Figure 1. Detailed geometric data can be found in Table 1. The shear test of the composite joint is conducted by applying the vertical load directly on the bolt, and the maximum load on the bolt will be taken as the ultimate shear load of the entire composite joint. Taking the D100-1/2H-M10-1 joint as an example, its sketch diagram is shown in Figure 1, and the physical diagram is shown in Figure 2.

## 3. Numerical Analysis and Verification

### 3.1. Geometric Model and Material Properties

**Figure 3:**
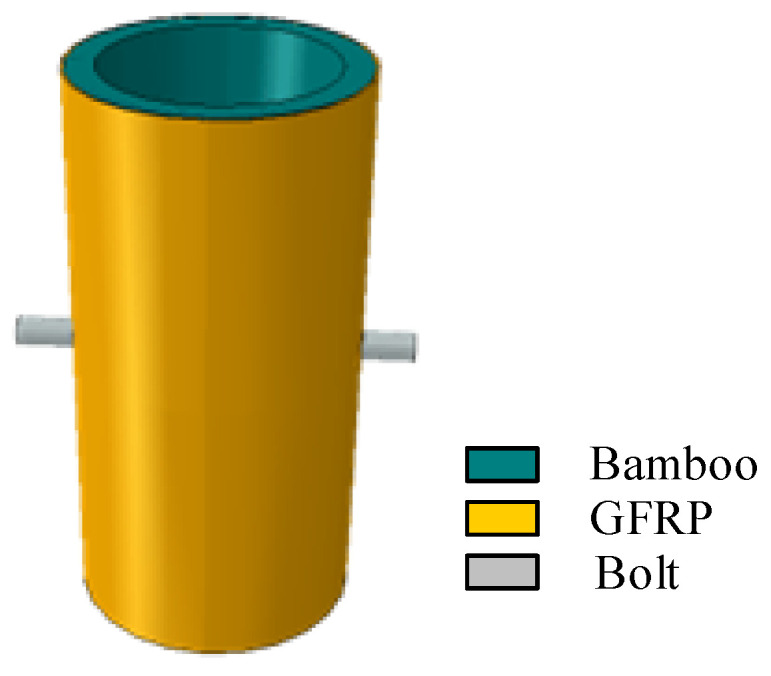
Geometric model of the composite joint.

With the case of the composite joint D100-1/2-M10-10, the geometric model for finite element calculation is established in ABAQUS according to the previous geometric data and loading mode, as shown in Figure 3. The bolt and bamboo tube are modeled as a three-dimensional solid element C3D8R, and the GFRP is modeled as a continuous shell element SC8R. Referring to Chen’s method [23], the GFRP is regarded as a macroscopic transverse isotropic material because it will be drilled with screw holes during the procession of the composite joint. The pressure along the longitudinal direction of the bamboo tube in the composite material can be reasonably assumed to be borne by epoxy resin, and the bearing capacity of the glass fiber is ignored. At the same time, continuous shell elements have the advantages of both shell elements and solid elements and are more accurate in contact calculations, which contributes to their extensive application in the numerical modeling of composite materials [24,25].

**Figure 4:**
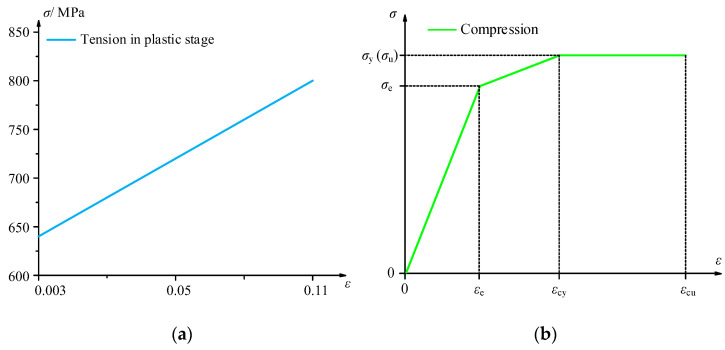
Stress–strain curve model of the bolt and bamboo. (**a**): Stress–strain curve model of bolt; (**b**): Stress–strain curve model of bamboo.

The constitutive relationship of grade 8.8 stud bolts and bamboo is shown in Figure 4 [26], and the bilinear function model used to characterize the stress–strain relation of steel bolts can be explained as follows:

(1) $$\sigma =\{\begin{array}{c} E_{{}_{s1}}\cdot \varepsilon \\ \sigma_{y}+E_{{}_{s2}}\cdot \left(\varepsilon -\varepsilon_{y}\right) \end{array}\begin{array}{c} 0\leq \varepsilon \leq \varepsilon_{y}\\ \varepsilon_{y}\leq \varepsilon \leq \varepsilon_{u} \end{array}$$

The parameter values of Formula (1) are described briefly as follows: *E*_*s*1_ = 210 GPa, *σ_y_* = 640 MPa, *σ_u_* = 800 MPa, and *ε_u_* = 0.103. The constitutive relationship of bamboo is measured according to *Testing Methods for the Physical and Mechanical Properties of Bamboo Used in Building* [27]. Referring to Wei’s model [20] and the mechanical test result, the threefold line model is adopted and described as follows:

(2) $$\sigma =\{\begin{array}{c} E_{{}_{1}}\cdot \varepsilon \\ \sigma_{e}+E_{{}_{2}}\cdot \left(\varepsilon -\varepsilon_{e}\right)\\ \sigma_{cy}=\sigma_{cu} \end{array}\begin{array}{c} 0\leq \varepsilon \leq \varepsilon_{e}\\ \varepsilon_{e}\leq \varepsilon \leq \varepsilon_{cy}\\ \varepsilon_{cy}\leq \varepsilon \leq \varepsilon_{cu} \end{array}$$

The values of the parameters in Formula (2) are as follows: *E*_1_ = 1.25 GPa, *E*_2_ = 0.15 *E*_1_, *σ_cu_* = 53.6 MPa, *ε_cu_* = 0.15, *ε_cy_* = 0.077, and *ε_e_* = 0.037. To obtain the mechanical properties of the GFRP combined with epoxy resin, tensile and shear tests were conducted based on *Fiber-Reinforced Plastics Composites-Determination of Tensile Properties* [28] and *Test Method for Longitudinal Transverse Shear (L-T Shear)* *Properties of Fiber-Reinforced Plastics* [29]. The test results are shown in Table 2. Based on the test results and relevant literature [30], the engineering elastic constants of the GFRP combined with the structural adhesive are selected in Table 3.

### 3.2. Meshing and Boundary Conditions

**Figure 5:**
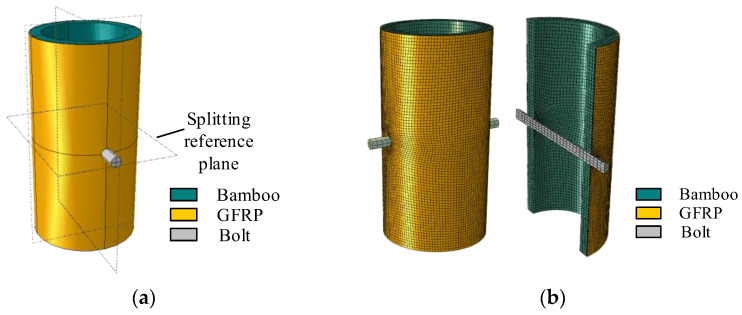
Division of the elements and components. (**a**) Simplified regional division; (**b**) Final element division.

**Figure 6:**
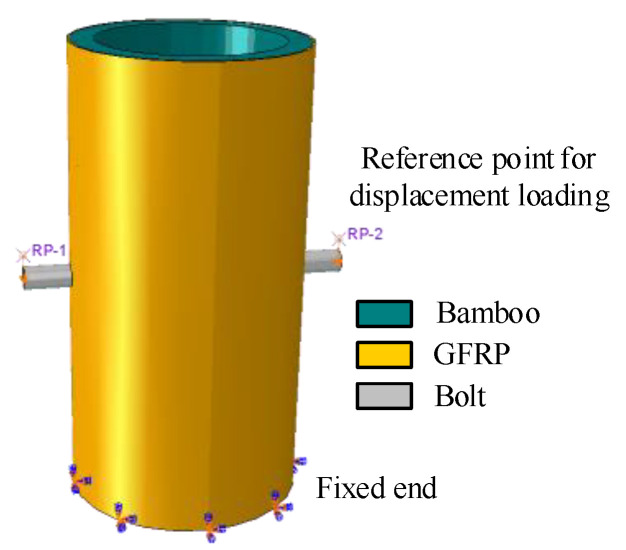
Boundary and loading conditions of simulation model.

Considering the symmetrical geometry, the composite joint D100-1/2-M10-10 was divided into eight parts by defining the datum plane centered on the screw hole. The specific partial division of the joint is shown in Figure 5a. More notably, the grid around the screw hole is thinly divided along the edge. The final meshing result is shown in Figure 5b. Considering the master–slave relationship of the contact surface and the calculation efficiency of the model, the mesh sizes in GFRP, bamboo and bolts are set as 2 mm × 2 mm, 3 mm × 3 mm, and 4 mm × 4 mm. The boundary area consists of the following four main contact surfaces: the bolt and bamboo tube wall, the bolt and GFRP, the GFRP and bamboo culm wall, and the fixed base and composite section composed of the bamboo culm and GFRP sheet. In the contact surface between the composite section and the fixed base, all directional displacements and rotation angles of the fixed end are constrained, as shown in Figure 6. The contact surface between the bolt and the bamboo culm wall and GFRP is defined as the “hard” contact in the normal direction and the “penalized” friction contact in the tangential direction with a coefficient of friction of 0.4 [26,31]. The contact surface between the GFRP and the bamboo culm outer wall is defined as a “tie” in ABAQUS because the GFRP and bamboo are tightly bonded by a structural adhesive without separation but with uniform radial displacement and limited vertical displacement [32,33].

### 3.3. Simulated Loading and Test Verification

**Figure 7:**
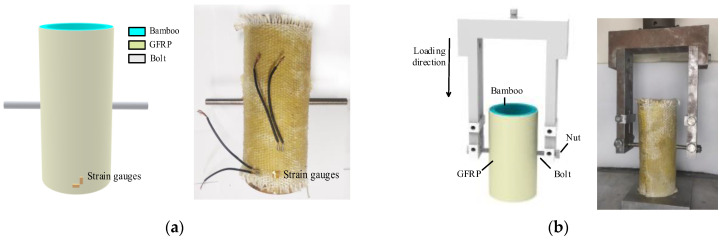
Position of the strain gauges and the loading method. (**a**) Position of strain gauges; (**b**) Loading method of composite joint.

As shown in Figure 6, the end faces of the bolts are all coupled to two reference points that are 10 mm above the center points, and the loading method applies the displacement on the reference point. Simultaneously, to verify the accuracy of the finite element model, an actual shear test of 3 composite joints in each group was conducted in this study. A set of 90° strain gauges were pasted on the outer surface of the GFRP lateral to the joint to collect the strain of the GFRP, whose specific location is shown in Figure 7.

## 4. Results and Discussion

### 4.1. Comparison of the Simulation Results and Test Results

#### 4.1.1. Stress Cloud Diagram and Failure Mode

**Figure 8:**
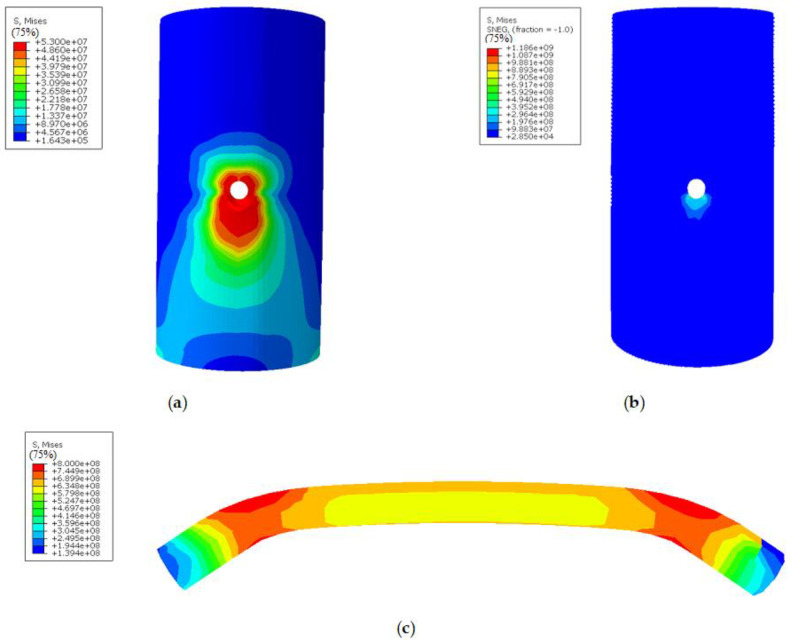
Stress nephogram of the components in the composite joint. (**a**) Stress nephogram of bamboo; (**b**) Stress nephogram of GFRP; (**c**) Stress nephogram of bolt.

**Figure 9:**
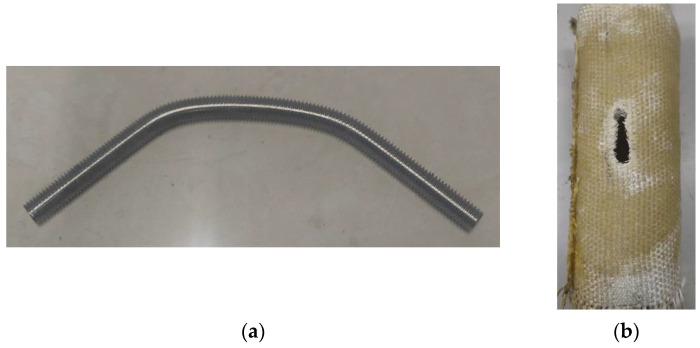
The failure mode of the components in the composite joint. (**a**) Failure mode of bolt; (**b**) Failure mode of bamboo wrapped with GFRP.

The failure mode of the other composite joints is similar to that of D100-0.5H-M10-1; thus, taking D100-0.5H-M10-1 as an example is reasonable. Moreover, the composite joints’ stress cloud diagram of each component is shown in Figure 8. The failure mode of each part of the composite joint in the test is shown in Figure 9.

From the stress cloud diagram obtained by the calculation model, the bamboo culm can be concluded to have reached the ultimate compressive strength at the screw hole. However, the compressive stress of the other parts is still low due to the constraint of the GFRP, while the GFRP has the maximum tensile stress at the longitudinal edge and bottom of the screw hole, which has exceeded the ultimate tensile strength. For the bolt, there are two plastic hinges near the screw hole, whose surface reaches the tensile strength. The pure bending section located inside the bamboo culm wall reaches the yield strength and enters the plastic stage.

According to the stress cloud diagram, the failure mode of the composite joint is speculated to be the local pressure-bearing failure of the bamboo culm. Fractures in the GFRP will gradually develop along the longitudinal edge and bottom of the screw hole, and the bolt will suffer bending failure near the screw hole. These results are in good agreement with the test failure of each component of the composite joint (Figure 9).

#### 4.1.2. Comparison between the Model and Specimen

**Figure 10:**
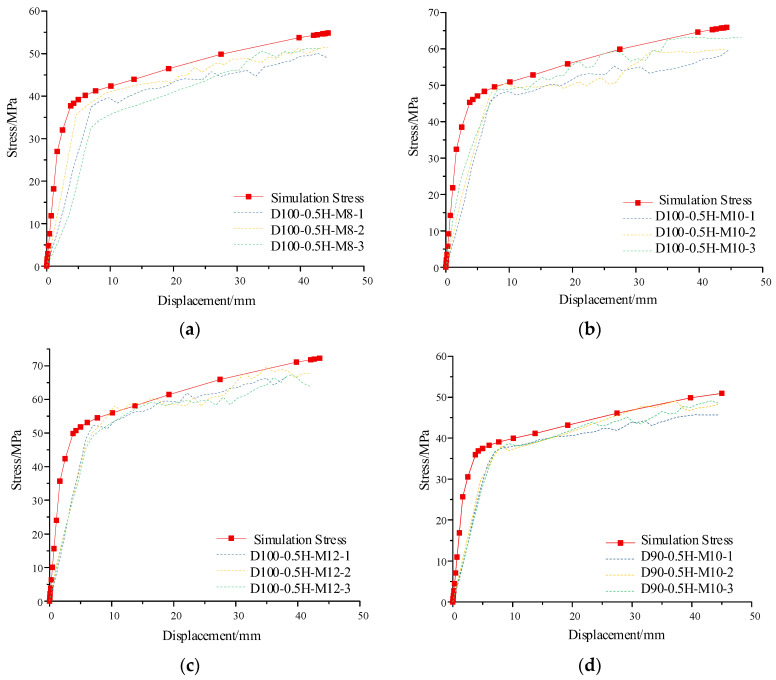

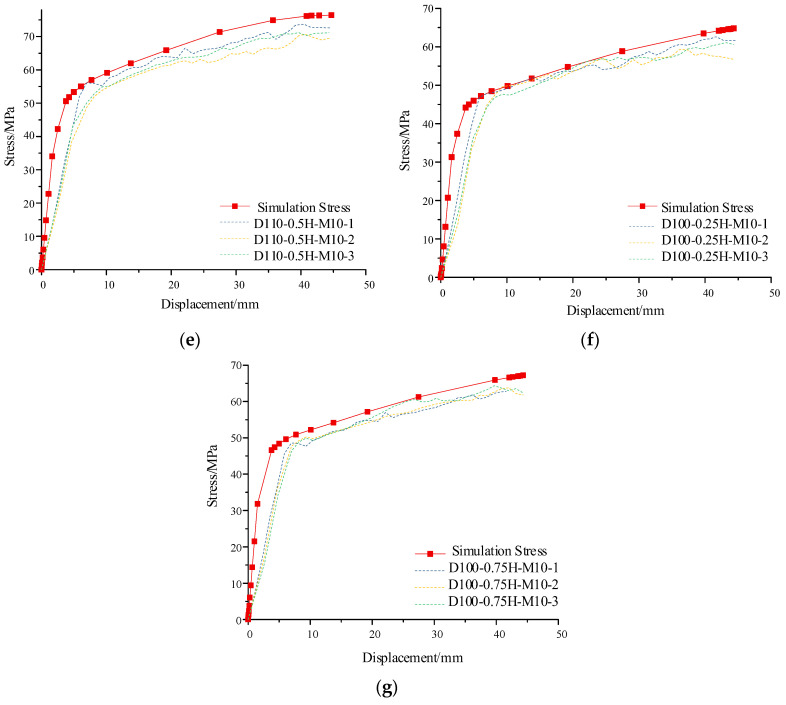
Comparison of simulated and test stress–displacement curves of composite joints. (**a**) Stress–displacement curves of D100-0.5H-M8; (**b**) Stress–displacement curves of D100-0.5H-M10; (**c**) Stress–displacement curves of D100-0.5H-M12; (**d**) Stress–displacement curves of D90-0.5H-M10; (**e**) Stress–displacement curves of D110-0.5H-M10; (**f**) Stress–displacement curves of D100-0.25H-M10; (**g**) Stress–displacement curves of D100-0.75H-M10.

**Figure 11:**
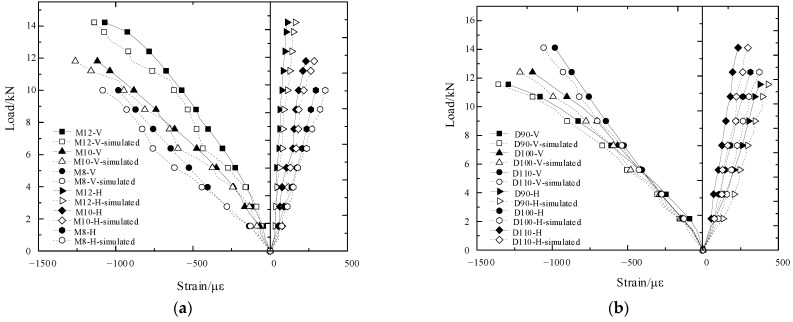

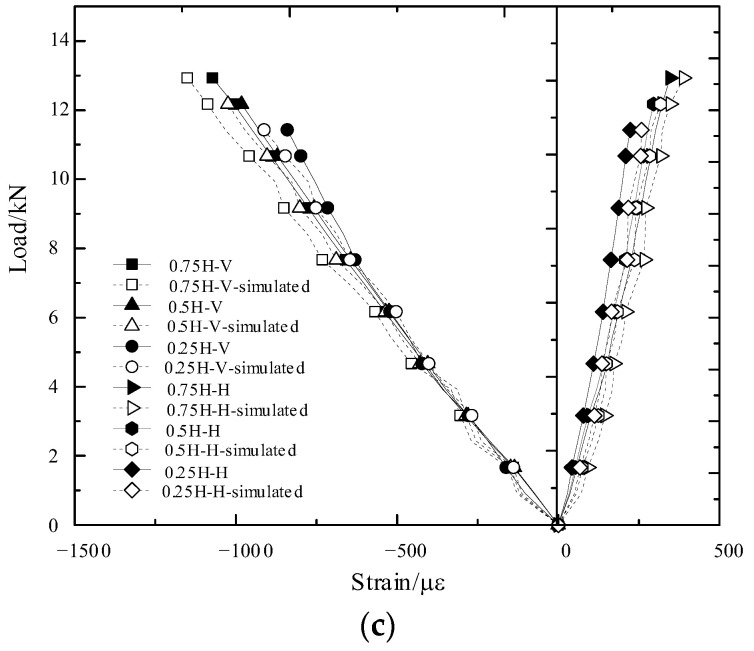
Comparison of the simulated and tested load–strain curves of the composite joints. (**a**) Load–strain curves under different bolt diameters; (**b**) Load–strain curves under different bamboo outer diameters; (**c**) Load–strain curves under different edge distances.

To reduce the interference in the comparison between the simulation curve and the test curve caused by the subtle size difference among the bamboo culms, the equivalent stress in the loading process is selected to characterize the shear bearing of the load on the composite joint. This equivalent stress (*σ*) is defined as the ratio of the shear load of the composite joint (*F*) to the effective stress area of the bolt hole (*A*). In the test, the effective compressed area (*A*) of the bolt hole equals the product of the sum wall thickness (Σ*t*_i_) of the bolt holes and the bolt diameter (*d*). The calculation formula of the above relationships is shown in Equation (3). The stress–displacement curves and the load–strain curves of the different composite joints are shown in Figure 10 and Figure 11. The maximum stress may be defined as the ultimate shear strength of the composite joint, and the test value is taken as the average value of the parallel specimens. In addition, the longitudinal and transversal strain of the first joint in the specimen group is selected as the test strain. The comparison between the test value and the simulated value is listed in Table 4.

(3) $$\{\begin{array}{c} \sigma =\frac{F}{A}\\ A=\sum t_{i}\cdot d \end{array}$$

All the curves contain two stages. Additionally, the following set of conclusions can be drawn according to the simulated and tested stress–displacement curves of the composite joints:

(1) Elastic linear growth stage:

At the beginning of the loading, each part of the joint is still in the elastic stage, the bamboo tube wall is compressed and deformed at the screw holes, and the bolts are bent. The maximum stress at this stage can reach approximately 70% of the joint shear strength.

(2) Elastic–plastic slow growth stage:

As the bending deformation of the bolts increases, the pure bending section of the bolts in the bamboo culm first enters the plastic stage, and the increase in the stress declines. At the same time, with the pressure-bearing failure of the bamboo culm, the bending failure of the bolts at the hole and the delamination tensile failure of the GFRP. The composite joint finally reaches the shear strength at this stage.

Comparing the simulated curve with the test curve, the initial stiffness of the test joint is smaller than that of the simulation model; however, in the elastic–plastic development stage, the initial stiffness is close to that of the simulation model. Furthermore, the bolt-hole contact in the test is not as perfect as that of the model. In addition, the test curve shows a fluctuating growth in the elastic–plastic developing stage, which indicates that the GFRP delamination failure mode has a certain influence on the stress or load of the test joint. However, the initial spacing between the bolt and hole has little influence on the final shear strength of the joint. According to the load–strain curve, with an increasing bolt diameter and bamboo culm outer diameter, the ultimate load of the composite joint increases, but the longitudinal and transversal strains of the outer GFRP decrease. Thus, the restraint effect of the GFRP is weakening. Compared to the bearing capacity loss caused by the decrease in the circumferential strain of the bamboo culm, the increase in the bearing load originates more from the expansion of the effective stress area of the screw hole and the effective restraint area of the GFRP. Moreover, circumferential strain increases, which is due to the effective restraint area of the GFRP on the top free section above the bolt. Thus, the increase in the end spacing of the bolt will lead to a slight rise in the bearing capacity of the composite joint. As shown in Figure 11, the simulated curve is close to the measured curve, which shows that the selection of the model is reasonable.

### 4.2. Parameter Analysis of the Simulation Model

**Figure 12:**
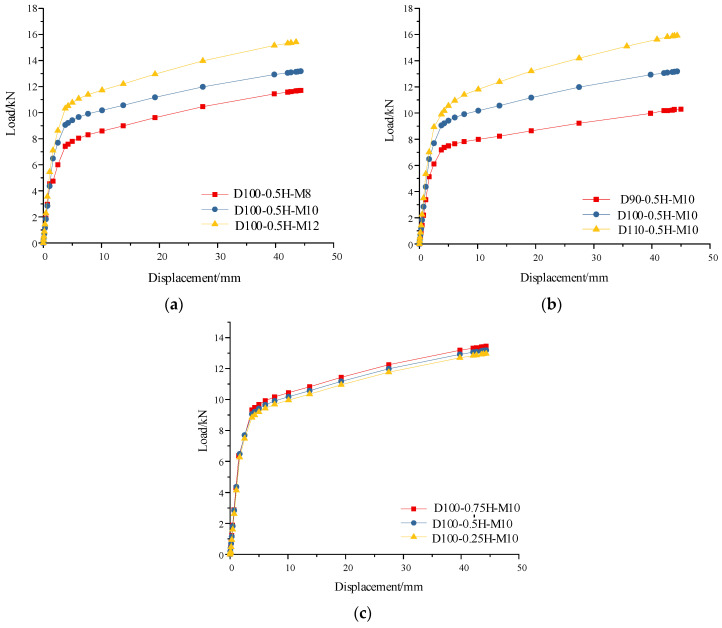
Effects of different structural parameters on bearing loads of the composite joints. (**a**) The effect of the bolt diameters on bearing load; (**b**) The effect of the bamboo outer diameters on bearing load; (**c**) The effect of the end space of the bolt on bearing load.

From the load–displacement curve of the calculation model, we can obtain the specific effects of the bolt diameter, bamboo tube outer diameter, and bolt end distance on the ultimate bearing capacity of the composite joints. As seen from Figure 12, the bolt diameter increases from 8 mm to 10 mm, and the ultimate load increases by 12.6%. When the bolt diameter increases from 10 mm to 12 mm, the ultimate load increases by 16.9%. The increase in the bolt diameter expands the contact area between the bolt and bamboo culm, which has a significant impact on the ultimate load of the composite joints. The larger the diameter is, the better the lifting effect of the bearing capacity. As the outer diameter of the bamboo culm increased from 90 mm to 100 mm, the ultimate load increased by 28.2%. When the outer diameter increased from 100 mm to 110 mm, the ultimate load increased by 20.8%. The increase in the bamboo culm outer diameter shortens the cantilever section of the bolt, which leads to a greater impact on the ultimate load of the composite joint than the bolt diameter. However, the corresponding lifting range decreases with an increasing outer diameter. When the end distance increases from 0.25 H to 0.5 H, the ultimate load increases by 2.1%. As the end distance increases from 0.5 H to 0.75 H, the ultimate load increases by 2.0%. After the composite joint t is strengthened by the FRP, the end distance of the bolt has little effect on the ultimate bearing capacity of the composite joints. As a result, the effect of the end distance does not need to be considered after being reinforced with the FRP.

## 5. Analytical Approaches

**Figure 13:**
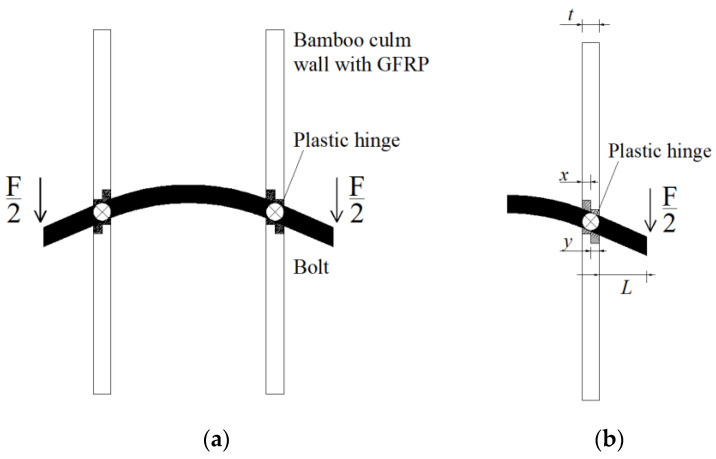
Load analysis mechanism of the composite joint. (**a**) Force diagram of the composite joint; (**b**) Semi-structural analysis diagram of the composite joint.

The load analysis is performed according to the double plastic hinge failure mode of the composite joint that was obtained from the previous numerical simulation calculation and actual test, as shown in Figure 13, and based on the bolt yield theory of Johansen [34] and the calculation model of the bamboo-bolt connection of GM. Oka [35] developed the calculation formula of the bearing capacity of the composite joint, which is derived as follows:

The force balance equation of the joint is as follows:

(4) $$\{\begin{array}{c} \frac{F_{y}}{2}=f_{b}\cdot (y-x)\\ t=x+y\\ M_{y}=\frac{F_{y}}{2}(L+y)-\frac{1}{2}f_{b}d\cdot y^{2} \end{array}$$

where *F_y_* is the bearing capacity of the composite joint (kN); *f_b_* is the compressive strength of bamboo; *t* is the average wall thickness of the bolt hole in the bamboo culm; *d* is the bolt diameter; *L* is the distance between the bamboo outer wall and loading end; and *M_y_* is the yield moment of the bolt. The parameter *x* is the distance between the inside wall of the bamboo and the plastic hinge, and *y* is the distance between the outside wall of the bamboo and the plastic hinge.

Finally, *F_y_* can be calculated as follows:

(5) $$F_{y}=f_{b}\cdot d\left[\sqrt{\frac{8M_{y}}{f_{b}\cdot d}+4L^{2}+t^{2}}-2L\right]$$

The calculation method of the yield moment of the bolt based on Eurocode 5 [36] is listed as follows:

(6) $$M_{y}=0.3f_{u}\cdot d^{2.6}$$

*M_y_*—the yield moment of the bolt, in N·mm;
*f_u_*—the tensile strength of the bolt, in N/mm^2^;
*d*—the diameter of the bolt, in mm.

The results in Table 5 show the analytical strength values according to Formulas (5) and (6) that correspond with the experimental values; the analytical strength value is the ratio of the analytical bearing load to the stress area of the bolt hole. According to Table 5, the analytical approach based on Eurocode 5 is not able to accurately describe the ultimate strength of the composite joints, as its error ratio will reach 20–30%. In addition, the influence of the end distance between the bolt and the pressure end of the bamboo culm is neglected in the analytical model. Significant errors originate from ignoring the GFRP and the wall thickness of bamboo culm; as a result, a coefficient of correction should be added to Formula (7) as follows:

(7) $$F_{y}=\alpha \cdot f_{b}\cdot d\left[\sqrt{\frac{8M_{y}}{f_{b}\cdot d}+4L^{2}+t^{2}}-2L\right]$$

*α* is the influence coefficient of the GFRP and wall thickness obtained by fitting the test value and analytical value of the composite joints. The value of the influence coefficient is 1.38 in this experiment.

After the correction, the error ratios of the analytical strength values are almost within 10%, which indicates that the revised analytical calculation method can correctly describe the bearing capacity of the composite joints. However, the error ratios of the simulated strength values are all less than 10% and close to 7% without any requirement for revision. The numerical model has higher accuracy, while the revised analytical model provides a simpler estimation method with a close result. Selecting the corresponding measure according to the local conditions is wise. Furthermore, the theoretical values of D90-0.5H-M10, along with the test values of D100-0.5H-M8 and D90-0.5H-M10, are all below the strength of bamboo, which indicates that a small bolt diameter and bamboo outer diameter will lead to an incomplete yield failure of bamboo in the composite joint. Therefore, when the FRP is used to wrap and strengthen the bamboo bolt composite joint, selecting bolts with a larger diameter and bamboo culm with a larger outer diameter can fully utilize the strength of the material. Finding that all the simulated strength values are higher than the test values is not difficult, which is due to the closer contact and more standard material strength in the numerical models of the composite joints.

## 6. Conclusions

The bamboo-bolt composite joint strengthened by the GFRP, which can be applied in modern bamboo structures, is investigated in this study. Numerical and analytical approaches were utilized to analyze the failure mode and ultimate shear load of the composite joints. The main conclusions are summarized as follows:

(1) The restraint effect of the FRP effectively prevents the occurrence of fracture along the grain of the bamboo tube in the composite joint, and the failure mode changes to a local bearing failure at the screw hole, which is a ductile failure that leads to full use of the material properties of bolts and bamboo. According to the tress cloud diagram and laboratory test results, the failure process begins with the bending deformation of the bolt, then the compression yield failure of the bamboo culm, and finally the layered tensile failure of the GFRP, which is accompanied by the partial plastic failure of the bolt at the screw hole.

(2) The ultimate bearing load of the composite joints is significantly influenced by the outer diameter of the bamboo and the bolt diameter, but the end distance of the bolt has little impact on the bearing capacity. In addition, the variables above will not change the ductile failure mode of the composite joints. When the bolt diameter increases by 2 mm, the ultimate load increases by 12.6–16.9%; an increase of 10 mm in the outer diameter of the bamboo culm will lead to an increase of 20.8–28.2% in the ultimate load. The small scale in the bolt diameter and the outer diameter of bamboo will lead to an incomplete yield failure of bamboo in the composite joints and insufficient utilization of the material.

(3) The numerical approach can correctly describe the experimental results, whose relative error is less than 10% and nearly 7%. However, the analytical method cannot describe the ultimate load of the composite joint because of the neglect of the restraint effect of the GFRP and the wall thickness of the bamboo culm. After the addition of the coefficient of correction in the analytical equation, the theoretical values are in good agreement. Therefore, a simpler analytical method is needed to be revised according to the fitting between the theoretical values and test values, and the error ratios of the analytical correction values are almost less than 10%. A comparison between the numerical and analytical results reveals that the load values in the numerical approach are in good agreement with that in the analytical method.

(4) In this work, a new way to enhance the shear bearing capacity of bamboo-bolt composite joint is to wrap bamboo culm wall with GFRP. The new composite joint has made full use of the material properties of bamboo and GFRP, which prevent the splitting failure mode in natural bamboo. Moreover, the FE model of composite joints are established through ABAQUS, and the simulation results agree well with actual test results and analytical results. The preliminary achieved results provide a beneficial reference in strengthening bamboo and wood structures. In further research, in order to optimize mechanical performance of composite joint, the impacts of number of FRP layers and bolts, along with types of FRP on ultimate shear load, will be investigated.

## Figures and Tables

**Table 1:** Geometric data of different composite joint models.

Outer Diameter/mm	End Distance	Screw Diameter/mm	Wall Thickness/mm
90	H/2	10	10
100	H/2	10	10
110	H/2	10	10
100	H/2	10	10
100	H/4	10	10
100	3H/4	10	10
100	H/2	8	10
100	H/2	12	10

**Table 2:** Mechanical properties of the GFRP combined with E51.

Tensile Elastic Modulus (*E*_t_)/GPa	Tensile Strength (*f*_t_)/MPa	Tensile Elastic Modulus (*G*)/GPa	Shear Strength (*f*_v_)/MPa	Elongation (*δ*)/(%)
14.1	173.5.	1.1	22.3	1.8

**Table 3:** Engineering elastic constants of the GFRP combined with E51.

E_1_/GPa	E_2_ = E_3_/GPa	G_12_ = G_13_/GPa	G_23_/GPa	Poisson Ratio
14	1.2	1.1	1	0.18

**Table 4:** Comparison of the simulated strength and test strength.

Group	Simulated Value/MPa	Test Value/MPa	Error/%
D100-0.5H-M8	54.83	53.95	1.63
D100-0.5H-M10	65.91	61.08	7.91
D100-0.5H-M12	72.24	68.01	6.22
D90-0.5H-M10	50.92	47.51	7.19
D110-0.5H-M10	76.42	70.49	8.42
D100-0.25H-M10	64.81	60.36	7.36
D100-0.75H-M10	67.21	62.58	7.40

**Table 5:** Comparison of the strength values of the composite joints in different approaches.

Specimen Group	Simulated Value /MPa	Analytical Value /MPa	Analytical Correction Value/MPa	Test Value /MPa	Simulated Error /%	Analytical Error /%	Analytical Correction Error /%
D100-0.5H-M8	54.83	32.00	44.16	50.95	7.63	−37.19	−13.32
D100-0.5H-M10	65.91	42.64	58.85	61.08	7.91	−30.19	−3.66
D100-0.5H-M12	72.24	53.80	74.25	68.01	6.22	−20.89	9.17
D90-0.5H-M10	50.92	36.05	49.75	47.51	7.19	−24.12	4.71
D110-0.5H-M10	76.42	51.60	71.21	70.49	8.42	−26.79	1.03
D100-0.25H-M10	64.81	41.76	57.63	60.36	7.36	−30.81	−4.52
D100-0.75H-M10	67.21	42.87	59.16	62.58	7.40	−31.50	−5.47

## Data Availability

The data that support the findings of this study are available from the corresponding author upon reasonable request.

## References

[1] Chen X., Xu Q.F., Harries K.A. (2015). Research on Mechanical Properties and Application of Bamboo in Civil Engineering: State-of-the-art. Struct. Eng..

[2] Zhou G.M., Jiang P.K. (2004). Density, Storage and Spatial Distribution of Carbon in *Phyllostachy pubescens* forest. Sci. Silvae Sin..

[3] Zhou Y.F., Gu L., Liu H.Z., Zhou G.M., Li C.Q., Shi Y.J., Han X., Lin H. (2013). Carbon Transfer During Manufacturing of Moso Bamboo Plank Using the Bamboo Unfolding and Flattening Technology. Sci. Silvae Sin..

[4] Liu J., Zhang J.L., Guo J., Li Y.S. (2013). The Development Status of the Modern Bamboo Structure Buildings. For. Eng..

[5] Zhou Y., Deng Y.S., Peng K., Wang L. (2018). Application of Bamboo Pipe Piles in Civil Engineering. J. Hubei Univ. Technol..

[6] Zhou J.W., Zhao F.H., Qi Y.S., Zhu J.Q., Chen Y.H. (2017). Experimental Research on Seismic Behavior of A New Fabricated Bamboo (Timber) Frame Connection. Ind. Constr..

[7] Zhang H.Z., Wang L.J., Yang T.L., Yang Y.G., Li H.Y. (2017). Theoretical Research on Nodes of Bamboo Structures under Simple Shear. Build. Struct..

[8] Yan Y.F., Chen Y.L., Wen Z.S., Zhou Q. (2014). The Exposition and Evaluation of the Modern Original Bamboo Structure Node Construction Logic. Archit. Technol..

[9] Morisco (1999). Yogyakarta: Nafiri Offset. Rekayasa Bambu..

[10] Hu H., Yang J., Wang F.L., Zhang Y.M. (2018). Mechanical Properties of Bolted Joints in Prefabricated Round Bamboo Structures. J. For. Eng..

[11] Feng P., Ye L.P., Meng X.M. Progress in the Study of FRP Strengthened Metallic Structures. Proceedings of the 22nd National Conference on Structural Engineering.

[12] Lu Y.Y., Huang Y.S., Zhang H.J., Liu L. (2006). New Progress in the Study of the Technology of Reinforcement with Fiber Reinforced Plastics. China Railw. Sci..

[13] Han J., Liu W.Q., Fang H. (2020). Application of fiber-reinforced resin matrix composites in the civil infrastructure field. J. Nanjing Technol. Univ. (Nat. Sci. Ed.).

[14] Perfetto D., Greco A., Caputo F. Experimental investigation of GFRP plates under LVI phenomena with different impact energy levels. Proceedings of the AIP Conference Proceedings.

[15] Davalos J.F., Zipfel M.G., Qiao P. (1999). Feasibility study of prototype GFRP-reinforced wood railroad crosstie. J. Compos. Constr..

[16] Nor N.M., Xin T.H., Yusof M.A. (2020). Enhancing Strength of Bamboo using GFRP and PU. Int. J. Sustain. Constr. Eng. Technol..

[17] Zhang Z., Meng X., Zhai J., Feng P. (2021). Experimental Study on Mechanical Properties of Bamboo Culms and Joints Reinforced with GFRP Sheets. International Conference on Fibre-Reinforced Polymer (FRP) Composites in Civil Engineering.

[18] Awaludin A., Andriani V. (2014). Bolted bamboo joints reinforced with fibers. Procedia Eng..

[19] Fu W.S., Zhao Z.R., Han W., Zhou J.B. (2014). Research on finite element model for parallel to bamboo culms axial shear. Appl. Mech. Mater..

[20] Wei Y., Yan S.C., Chen S., Duan M.J., Wang L.B. (2019). Numerical simulation on bending performance of FRP reinforced bamboo beams. Acta Mater. Compos. Sin..

[21] Li J. (2014). Experimental Study on Mechanical Properties of Stirrup Enhanced Glued Bamboo Columns. Master’s Thesis.

[22] Perfetto D., Lamanna G., Sepe R., Luca A.D. (2020). Design of a Bamboo Treadmill Bicycle Main Frame. Macromol. Symp..

[23] Chen Z.H., Luo Q.W. (2019). Numerical simulation of mechanical properties of gfrp-concrete composite beam with consideration of interface bond-slip. Ind. Constr..

[24] Thorsson S.I., Waas A.M., Rassaian M. (2018). Low-velocity impact predictions of composite laminates using a continuum shell based modeling approach part A: Impact study. Int. J. Solids Struct..

[25] Schwarze M., Vladimirov I.N., Reese S. (2010). A new continuum shell finite element for sheet metal forming applications. Int. J. Mater. Form..

[26] Hu H. (2018). Research on Bolt Joint’s Mechanical Properties of Prefabricated Bamboo Structure. Master’s Thesis.

[27] Ministry of Construction of the PRC (2007). Testing Methods for Physical and Mechanical Properties of Bamboo Used in Building.

[28] Standardization Administration of China (2005). Fiber-Reinforced Plastics Composites-Determination of Tensile Properties.

[29] Standardization Administration of China (2005). Test Method for Longitudinal Transverse Shear (L-T Shear) Properties of Fiber Reinforced Plastics.

[30] Jiao G.Q., Jia P.R. (2008). Mechanics of Composite Materials.

[31] Eid Alajmi A., Alotaibi J.G., Yousif B.F., Nirmal U. (2021). Tribological studies of bamboo fibre reinforced epoxy composites using a BOD technique. Polymers.

[32] Song S.Z., Wei J.J., Chen C. (2015). A study on the performance of tubular T-Joints strengthend with CFRP subjected to monotonic loading. Prog. Steel Build. Struct..

[33] Luo J.H. (2019). Experimental Study and Numerical Simulation of CFRP Reinforced Compression Square Steel Pipe. Master’s Thesis.

[34] Johansen K.W. (1949). International Association of Bridge and Structural Engineering. Theory Timber Connect..

[35] Oka G.M., Triwiyono A., Awaludin A., Siswosukarto S. (2015). Experimental and theoretical investigation of bolted bamboo joints without void filled material. Appl. Mech. Mater..

[36] BSI (2004). Design of Timber Structures-Part 1-1: General-Common Rules and Rules for Buildings: BS EN 1995-1-1, Eurocode 5.

